# The Influence of Climate Warming and Humidity on Plant Diversity and Soil Bacteria and Fungi Diversity in Desert Grassland

**DOI:** 10.3390/plants10122580

**Published:** 2021-11-25

**Authors:** Yi Zhang, Yingzhong Xie, Hongbin Ma, Juan Zhang, Le Jing, Yutao Wang, Jianping Li

**Affiliations:** 1 College of Agriculture, Ningxia University, Yinchuan 750021, China; 13995378736@163.com (Y.Z.); xieyz@nxu.edu.cn (Y.X.); ma_hb@nxu.edu.cn (H.M.); 18435121531@163.com (J.Z.); jinglevip@163.com (L.J.); 18234171762@163.com (Y.W.); 2 State Key Laboratory Cultivation Base for Northwest Degraded Ecosystem Recovery and Reconstruction, Yinchuan 750021, China

**Keywords:** precipitation changes, increased temperatures, desert grassland, plant diversity, plant biomass, soil bacteria diversity, soil fungi diversity

## Abstract

Our study, which was conducted in the desert grassland of Ningxia in China (E 107.285, N 37.763), involved an experiment with five levels of annual precipitation 33% (R33), 66% (R66), 100% (CK), 133% (R133), 166% (R166) and two temperature levels (inside Open-Top Chamber (OTC) and outside OTC). Our objective was to determine how plant, soil bacteria, and fungi diversity respond to climate change. Our study suggested that plant *α*-diversity in CK and TCK were significantly higher than that of other treatments. Increased precipitation promoted root biomass (RB) growth more than aboveground living biomass (ALB). R166 promoted the biomass of Agropyron mongolicum the most. In the fungi communities, temperature and precipitation interaction promoted *α*-diversity. In the fungi communities, the combination of increased temperature and natural precipitation (TCK) promoted *β*-diversity the most, whose distance was determined to be 25,124 according to PCA. In the bacteria communities, *β*-diversity in CK was significantly higher than in other treatments, and the distance was determined to be 3010 according to PCA. Soil bacteria and fungi *α*- and *β*-diversity, and ALB promoted plant diversity the most. The interactive effects of temperature and precipitation on C, N, and P contents of plants were larger than their independent effects.

## 1. Introduction

One of the most severe environmental problems facing mankind is undoubtedly climate change, which is dominated by climate warming [1]. During 1982–2012, high latitudes experienced greater temperature increases than middle latitudes, land temperatures increased faster than ocean temperatures, and the average global temperature increased by 0.85 °C compared with the same time period (1981–2017). In the context of global climate change, extreme weather events frequently occur, and precipitation is unevenly distributed [2].

Vegetation is the link connecting soil, the atmosphere, and water. As an important part of the terrestrial ecosystem, it plays an “indicator” role in global climate change. The vegetation index reflects surface vegetation characteristics and vegetation cover information [3]. Therefore, to a large extent it represents the ecological quality of a certain area [4].

Plant biodiversity includes four components: genetic diversity, species diversity, intraspecific genetic diversity, and ecosystem diversity. Species diversity is the manifestation of biodiversity in species. It is a simple measure of biodiversity [5], counting only the number of different species in a given area. Plant species diversity is the basic unit that constitutes a plant community and forms the core part of biodiversity. Plant species diversity mainly includes plant species composition, richness, uniformity, interspecies relationships, and their changes in time and space [6]. A sustained high level of plant diversity can improve ecosystem stability and its resistance to changes in habitat. Plant species diversity is an important indicator for analyzing the structure, function, succession, and ecological restoration stages of plant communities [7].

Species alpha (*α*) diversity refers to the diversity of species in the same location or community. It is caused by differences in niches among species [8]. Its measurement is divided into four categories: species richness, relative abundance models of species, the ecological diversity index, and the evenness index [9]. Biodiversity is reflected by plant diversity and its functional characteristics, and it can also explain the interaction between vegetation and the environment [10]. The composition and development of plant communities can be well understood by studying biodiversity. This work has theoretical and practical significance for maintaining a greater variety of organisms [11]. Plant community diversity generally refers to differences in composition, structure, function, and dynamics of plant communities and is a foundational level of research among all levels of biodiversity [12]. In biodiversity conservation practice, the status of communities or ecosystems is often evaluated based on the diversity index. The measurement of community alpha diversity can be divided into four categories: (1) the Shannon–Wiener diversity index reflects the hierarchical characteristics of a biological community; the higher the value, the higher the community diversity; (2) the Margalef species richness index reflects the number of plant species in the community; (3) the Pielou evenness index refers to the distribution ratio of the number of individuals in the community; (4) the Simpson dominance index reflects the distribution of numerically abundant species within a community. The four commonly used *α*-diversity indexes incorporate two measurements—the number (richness) of species and the uniformity of species [13].

Soil microbes degrade and detoxify environmental pollutants and play an important role in maintaining soil quality and ecosystem stability [14]. The diversity and variability within the soil microorganism community reflects its diverse responses and adaptations to the environment [15].

As an important part of the soil, soil microorganisms are often hailed as the “converter” of nutrient cycling, the “regulator” of terrestrial ecosystem stability, and the “purifier” of environmental pollution [16]. The soil microorganism community can be used to monitor changes in the structure and function of grassland ecosystems following fluctuations in water availability. Changing precipitation patterns have been one of the hot issues in global climate research in recent years, and grassland ecosystems are widely distributed, with most of them located in ecologically fragile zones [17]. They are susceptible to disturbance and global climatic imbalances, especially those in areas with restricted water such as arid and semi-arid grassland ecosystems. Soil microorganisms act as catalysts for soil nutrient cycling and transformation, which can promote absorption [18].

We conducted a study in the Ningxia Hui Autonomous Region of China. We used an Open-Top Chamber (OTC) to simulate increased temperature and artificial shelters and sprinklers to simulate precipitation changes. We systematically studied the changes in temperature and precipitation and the interaction of the two factors:

(i) Dynamic changes with regards to about the *α*-diversity, biomass, organic carbon, total nitrogen, and total phosphorus of plants in the desert steppe ecosystem;
(ii) Dynamic changes with regards to the *α*- and *β*-diversity of the soil fungi and soil bacteria;
(iii) The synergistic relationship between the *α*-diversity, biomass, organic carbon, total nitrogen, total phosphorus of plants, and the *α*- and *β*-diversity of the soil fungi and soil bacteria. The research results provide a reliable theoretical basis for the formulation of reasonable response strategies for desert grasslands.

## 2. Results

### 2.1. Plant Importance Value

**Figure 1:**
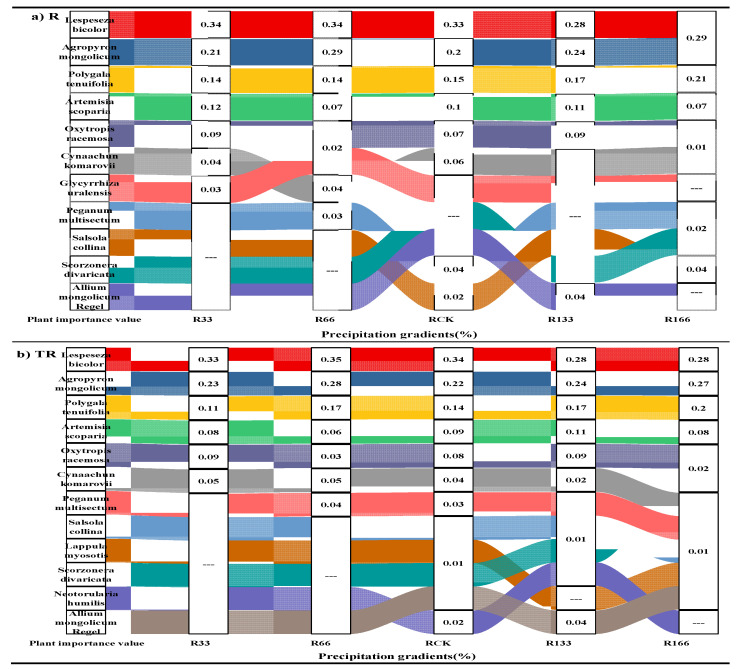
The main values of plants in the study sites. (**a**) The main values of plants under precipitation changing treatment (R); (**b**) The main values of plants under the interaction of precipitation changing and temperature increasing treatment (TR). Five levels of rainfall (R) were used: 33% (R33), 66% (R66), 100% (CK), 133% (R133), and 166% (R166) of the annual average. The first two rainfall conditions were obtained by using two rainout shelters with two manipulated rainfall doses: 97 mm (R33) and 194 mm (R66). For the three other rainfall conditions, we artificially increased rainfall pot in unsheltered plots using a watering: 295 mm (CK), 392 mm (R133), and 490 mm (R166). The temperature consisted of two levels: the actual temperature (CK) and the interaction between rainfall and the temperature, which was increased by about 2 °C (T) with the OTC (Open-Top Chamber) in each plot. TR33 was the first site of interaction between 33% precipitation (R33) and the temperature increase of about 2 °C (T); the marks of TR66, TCK, TR133, TR166 were the same. R33 was the first site of 33% precipitation, and the marks of R66, CK, R133, R166 were the same.

Under the changing precipitation condition and the interaction of the precipitation changed and the temperature increased, the main value of the Agropyron mongolicum, Lespedeza bicolor, and Polygala tenuifolia were all higher than the other plants (Figure 1).

### 2.2. Plant α-Diversity

The plant *α*-diversity had no significant differences among the different precipitation and temperature treatments (Table 1).

### 2.3. The Number of Species

**Figure 2:**
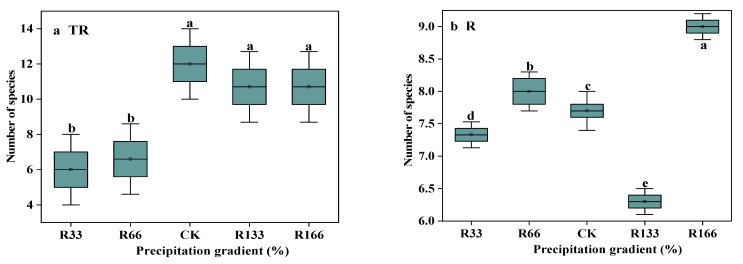

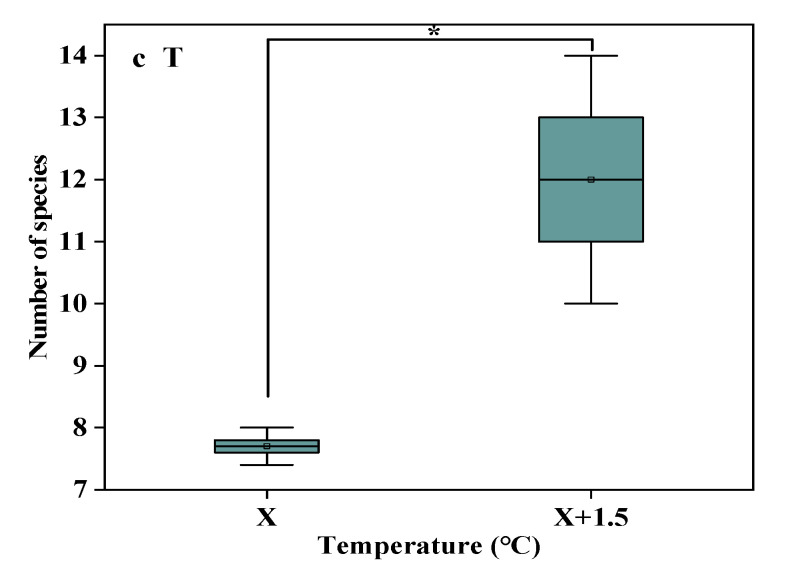
The number of species in the study sites. (**a**) The number of species under precipitation changing treatment (R); (**b**) The number of species under interaction of precipitation changing and temperature increasing treatment (TR). (**c**) The number of species under temperature increasing treatment (T). Five levels of rainfall (R) were used: 33% (R33), 66% (R66), 100% (CK), 133% (R133), and 166% (R166) of the annual average. The first two rainfall conditions were obtained by using two rainout shelters with two manipulated rainfall doses: 97 mm (R33) and 194 mm (R66). For the three other rainfall conditions, we artificially increased rainfall in unsheltered plots using a watering pot: 295 mm (CK), 392 mm (R133), and 490 mm (R166). The temperature consisted of two levels: the actual temperature (CK) and the interaction between rainfall and the temperature, which was increased by about 2 °C (T) with the OTC (Open-Top Chamber) in each plot. TR33 was the first site of interaction between 33% precipitation (R33) and the temperature increase of about 2 °C (T), R33 was the first site of 33% precipitation, and other marks were the same. Values indicate the mean ± SE; different letters represent a significant difference according to LSD test (*p* < 0.05). * represents a significant difference according to *t*-test (*p* < 0.05).

Under the interaction of rising precipitation levels and increasing temperature conditions, the number of species was highest in CK, which was significantly higher than R33 (*p* < 0.05). With increased precipitation, the number of species was highest under R166, which was significantly higher than other precipitation treatments (*p* < 0.05). The difference between the natural temperature and the temperature increases was obvious (*p* < 0.05) (Figure 2).

### 2.4. The Biomass of Plants and Dominant Plant Species

**Figure 3:**
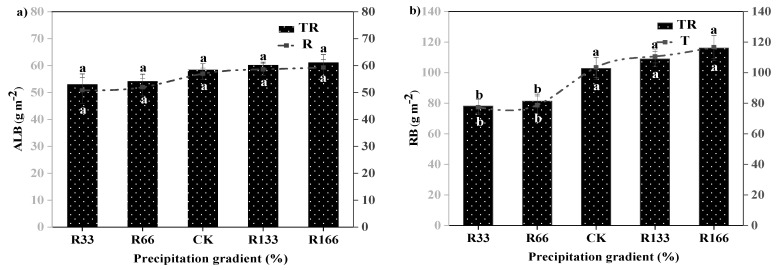

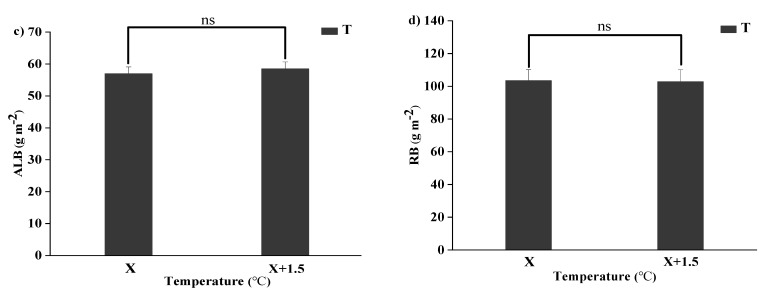
Variations of aboveground plant living biomass (ALB) and plant root biomass (RB) of vegetation in the study sites. (**a**) Aboveground plant living biomass (ALB) under precipitation changing (R) and the interaction of the precipitation changing and temperature increasing (TR). (**b**) Root biomass (RB) under precipitation changing (R) and the interaction of the precipitation changing and temperature increasing (TR). (**c**) Aboveground plant living biomass (ALB) under temperature increasing (T). (**d**) Root biomass (RB) under temperature increasing (T). Five levels of rainfall (R) were used: 33% (R33), 66% (R66), 100% (CK), 133% (R133), and 166% (R166) of the annual average. The first two rainfall conditions were obtained by using two rainout shelters with two manipulated rainfall doses: 97 mm (R33) and 194 mm (R66). For the three other rainfall conditions, we artificially increased rainfall in unsheltered plots using a watering pot: 295 mm (CK), 392 mm (R133), and 490 mm (R166). The temperature consisted of two levels: the actual temperature (CK) and the interaction between rainfall and the temperature increased by about 2 °C (T) with the OTC (Open-Top Chamber) in each plot. TR33 was the first site of interaction between 33% precipitation (R33) and the temperature, which was increased by about 2 °C (T), and the marks of TR66, TCK, TR133, TR166 are the same. R33 was the first site of 33% precipitation, and the marks of R66, CK, R133, R166 were the same. Values indicate the mean ± SE, and different letters represent a significant difference according to LSD test (*p* < 0.05). ns represents a no significant difference according to *t*-test (*p* < 0.05).

The RB was significantly higher than the ALB. The differences of the ALB were not significant under the interaction of the changed precipitation and increased temperature conditions (*p* < 0.05). The root biomass was highest in R166 but lowest in R33, and their difference were very significant *(p* < 0.05). The difference of the ALB under the natural temperature and the increased temperature were not significant, the same as the RB (*p* < 0.05) (Figure 3).

**Figure 4:**
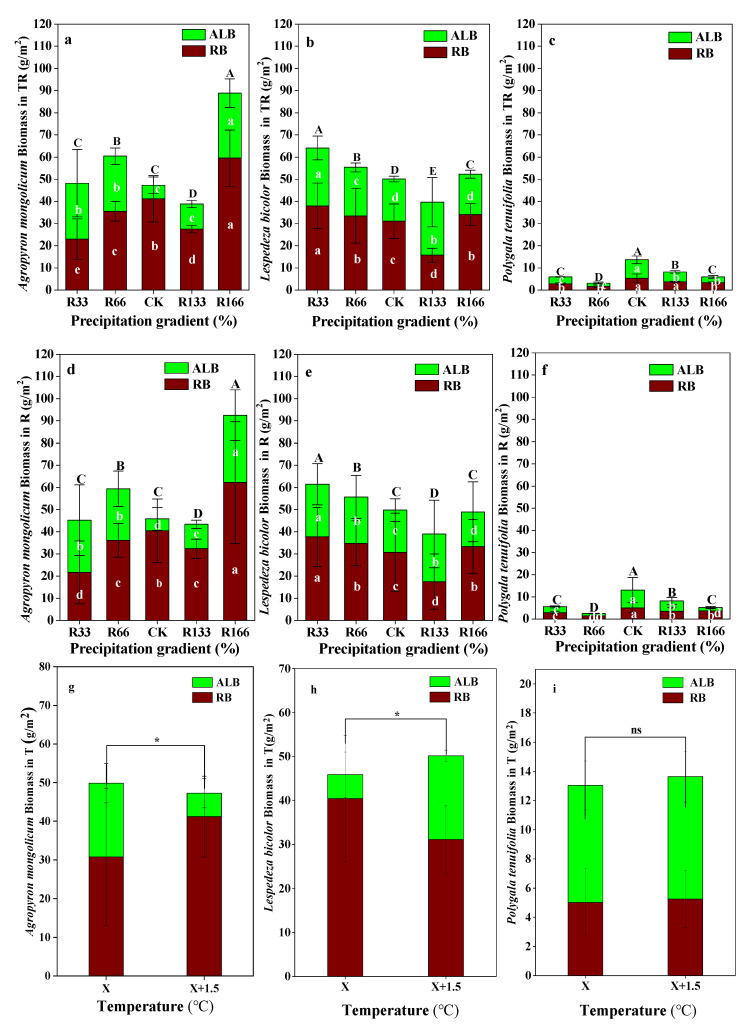
Variations of aboveground plant living biomass (ALB) and plant root biomass (RB) of dominant species in the study sites. (**a**) Aboveground plant living biomass (ALB) and plant root biomass (RB) of Agropyron mongolicum under the precipitation changing and temperature increasing(TR); (**b**) Aboveground plant living biomass (ALB) and plant root biomass (RB) of Lespedeza bicolor under the precipitation changing and temperature increasing(TR); (**c**) Aboveground plant living biomass (ALB) and plant root biomass (RB) of Polygala tenuifolia under the precipitation changing and temperature increasing(TR); (**d**) Aboveground plant living biomass (ALB) and plant root biomass (RB) of Agropyron mongolicum under the precipitation changing (R); (**e**) Aboveground plant living biomass (ALB) and plant root biomass (RB) of Lespedeza bicolor under the precipitation changing (R); (**f**) Aboveground plant living biomass (ALB) and plant root biomass (RB) of Polygala tenuifolia under the precipitation changing (R); (**g**) Aboveground plant living biomass (ALB) and plant root biomass (RB) of Agropyron mongolicum under the temperature increasing (T); (**h**) Aboveground plant living biomass (ALB) and plant root biomass (RB) of Lespedeza bicolor under the temperature increasing (T); (**i**) Aboveground plant living biomass (ALB) and plant root biomass (RB) of Polygala tenuifolia under the temperature increasing (T). Five levels of rainfall (R) were used: 33% (R33), 66% (R66), 100% (CK), 133% (R133), and 166% (R166) of the annual average. The first two rainfall conditions were obtained by using two rainout shelters with two manipulated rainfall doses: 97 mm (R33) and 194 mm (R66). For the three other rainfall conditions, we artificially increased rainfall in unsheltered plots using a watering pot: 295 mm (CK), 392 mm (R133), and 490 mm (R166). The temperature consisted of two levels: the actual temperature (CK) and the interaction between rainfall and the temperature increased by about 2 °C (T) with the OTC (Open-Top Chamber) in each plot. TR33 was the first site of interaction between 33% precipitation (R33) and the temperature, which was increased by about 2 °C (T), and the marks of TR66, TCK, TR133, TR166 were the same. R33 was the first site of 33% precipitation, and the marks of R66, CK, R133, R166 were the same. Values indicate the mean ± SE; different letters represent a significant difference according to LSD test (*p* < 0.05). ns represents a nonsignificant difference according to *t*-test (*p* < 0.05).

When precipitation was increased, Agropyron mongolicum had the highest RB under R166, and had the lowest RB under R33, the RB difference between the precipitation gradients was significant (*p* < 0.05). The ALB of Agropyron mongolicum was highest under the R166, which was significantly higher than other precipitation treatments, the same as the total biomass of Agropyron mongolicum (*p* < 0.05). The RB, ALB, and total biomass of Lespedeza bicolor were highest under R33, which were significantly higher than other precipitation gradients (*p* < 0.05). The RB, ALB, and total biomass of Polygala tenuifolia were the highest under natural precipitation, which were significantly higher than other precipitation treatments (*p* < 0.05) This phenomenon with regards to the Agropyron mongolicum, Lespedeza bicolor, and Polygala tenuifolia under the warming and precipitation interaction was the same (*p* < 0.05). With the increases temperature, the differences of the RB, ALB, and total biomass of Agropyron mongolicum and Lespedeza bicolor were significant, but the temperature increases had no obvious impact on the Polygala tenuifolia *(p* < 0.05) (Figure 4).

### 2.5. The Organic Carbon, Total Nitrogen, and Total Phosphorus of Plants and Dominant Plant Species

**Figure 5:**
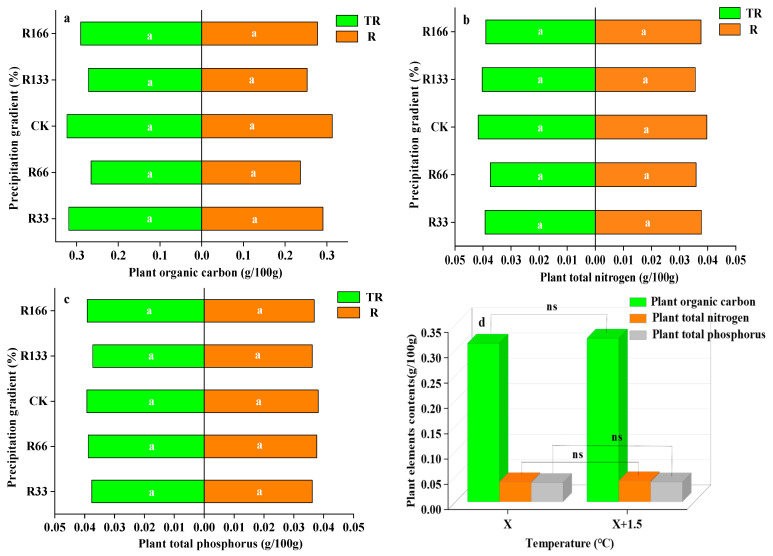
Percentages of plant organic carbon, plant total nitrogen, and total phosphorus of vegetation in the study sites. (**a**) Plant organic carbon under precipitation changing (R) and the interaction of the precipitation changing and temperature increasing (TR); (**b**) Plant total nitrogen under precipitation changing (R) and the interaction of the precipitation changing and temperature increasing (TR); (**c**) Plant total phosphorus under precipitation changing (R) and the interaction of the precipitation changing and temperature increasing(TR); (**d**) Plant organic carbon, plant total nitrogen, plant total phosphorus under temperature increasing (T). Five levels of rainfall (R) were used: 33% (R33), 66% (R66), 100% (CK), 133% (R133), and 166% (R166) of the annual average. The first two rainfall conditions were obtained by using two rainout shelters with two manipulated rainfall doses: 97 mm (R33) and 194 mm (R66). For the three other rainfall conditions, we artificially increased rainfall in unsheltered plots using a watering pot: 295 mm (CK), 392 mm (R133), and 490 mm (R166). The temperature consisted of two levels: the actual temperature (CK) and the interaction between rainfall and the temperature, which was increased by about 2 °C (T) with the OTC (Open-Top Chamber) in each plot. TR33 is the first site of interaction between 33% precipitation (R33) and the temperature increase of about 2 °C (T), and the marks of TR66, TCK, TR133, TR166 were the same. R33 was the first site of 33% precipitation, and the marks of R66, CK, R133, R166 were the same. Values indicate the mean ± SE, and different letters represent a significant difference according to LSD test (*p* < 0.05). ns represents a nonsignificant difference according to *t*-test.

The differences in plant organic carbon, plant nitrogen, and plant phosphorus content were not significant under the changing precipitation condition and the interaction of the changing precipitation and increasing temperature conditions (*p* < 0.05), but the plant organic carbon, plant nitrogen, and plant phosphorus content under the precipitation condition changed less than under the interaction of the temperature and precipitation conditions. With the increases in temperature, the differences of the plant organic carbon, plant total nitrogen, and plant total phosphorus were also not obvious (*p* < 0.05) (Figure 5).

**Figure 6:**
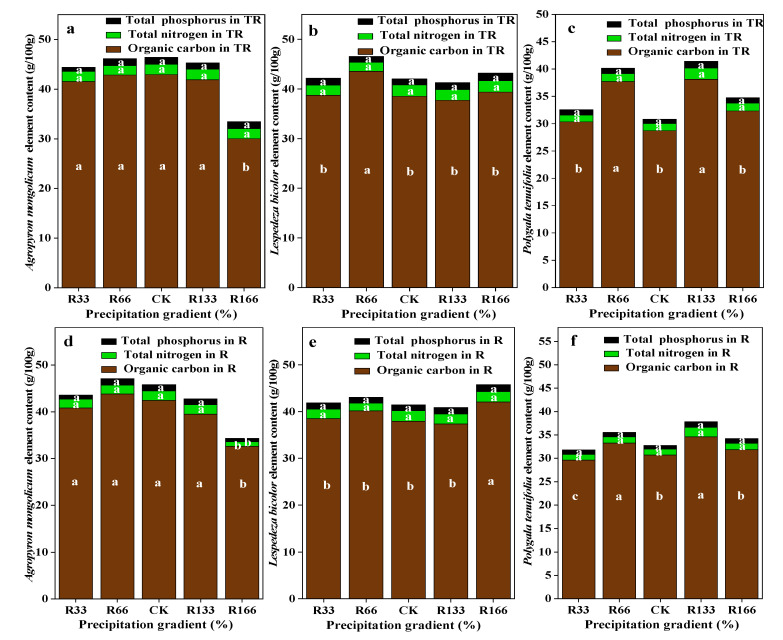

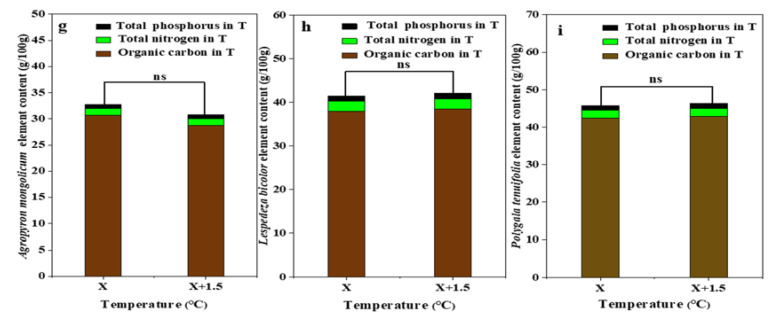
Percentage of plant organic carbon, plant total nitrogen, and total phosphorus of dominant species in the study sites. (**a**) The organic carbon, total nitrogen, total phosphorus of Agropyron mongolicum under the interaction of the precipitation changing and temperature increasing (TR); (**b**) The organic carbon, total nitrogen, total phosphorus of Lespedeza bicolor under the interaction of the precipitation changing and temperature increasing(TR); (**c**) The organic carbon, total nitrogen, total phosphorus of Polygala tenuifolia under the interaction of the precipitation changing and temperature increasing(TR); (**d**) The organic carbon, total nitrogen, total phosphorus of Agropyron mongolicum under the precipitation changing (R); (**e**) The organic carbon, total nitrogen, total phosphorus of Lespedeza bicolor under the precipitation changing (R); (**f**) The organic carbon, total nitrogen, total phosphorus of Polygala tenuifolia under the precipitation changing (R); (**g**) The organic carbon, total nitrogen, total phosphorus of Agropyron mongolicum under the temperature increasing (T); (**h**) The organic carbon, total nitrogen, total phosphorus of Lespedeza bicolor under the temperature increasing (T); (**i**) The organic carbon, total nitrogen, total phosphorus of Polygala tenuifolia under the temperature increasing (T). Five levels of rainfall (R) were used: 33% (R33), 66% (R66), 100% (CK), 133% (R133), and 166% (R166) of the annual average. The first two rainfall conditions were obtained by using two rainout shelters with two manipulated rainfall doses: 97 mm (R33) and 194 mm (R66). For the three other rainfall conditions, we artificially increased rainfall in unsheltered plots using a watering pot: 295 mm (CK), 392 mm (R133), and 490 mm (R166). The temperature consisted of two levels: the actual temperature (CK) and the interaction between rainfall and the temperature, which was increased by about 2 °C (T) with the OTC (Open-Top Chamber) in each plot. TR33 was the first site of interaction between 33% precipitation (R33) and the temperature increase of about 2 °C (T), and the marks of TR66, TCK, TR133, TR166 are the same. R33 was the first site of 33% precipitation, and the marks of R66, CK, R133, R166 are the same. Values indicate the mean ± SE, and different letters represent a significant difference according to LSD test (*p* < 0.05).

For the interaction of the temperature and precipitation conditions, plant organic carbon was the lowest at R166 for *Agropyron mongolicum*, which was significantly lower than other treatments (*p* < 0.05). Plant organic carbon was the highest at R66 for Lespedeza bicolor and was highest at R133 for Polygala tenuifolia, which were both significantly higher than other treatments (*p* < 0.05). Plant total nitrogen and plant total phosphorus were not significant among the different treatments (*p* < 0.05). The plant organic carbon, total nitrogen, and total phosphorus for *Agropyron mongolicum*, Lespedeza bicolor, and Polygala tenuifolia at the precipitation changed was lower. With the increased temperature, the differences of plant organic carbon, plant total nitrogen, and plant total phosphorus for *Agropyron mongolicum*, *Lespedeza bicolor*, and *Polygala tenuifolia* were not significant (*p* < 0.05) (Figure 6).

### 2.6. Soil Microorganism α-Diversity

In the fungi communities, *α*-diversity gradually decreased under the interaction of the increasing temperature and precipitation conditions. However, when only precipitation increased, *α*-diversity first decreased and then increased.

In the bacteria communities, under the control of the increasing temperature and precipitation conditions, the *α*-diversity did not show any obvious patterns.

**Figure 7:**
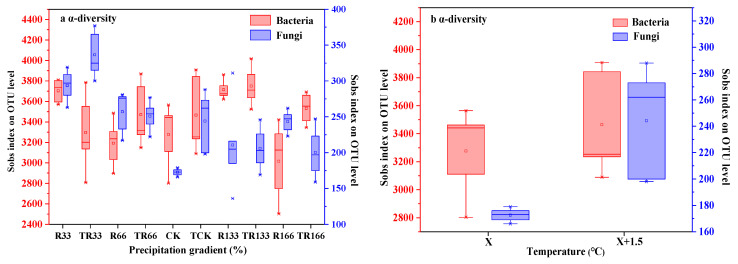
Soil microbial *α*-diversity of (**a**) fungi and (**b**) bacteria in the study sites by principal component analysis (PCA). Sobs index was the observed richness. Five levels of rainfall (R) were used: 33% (R33), 66% (R66), 100% (CK), 133% (R133), and 166% (R166) of the annual average. The first two rainfall conditions were obtained by using two rainout shelters with two manipulated rainfall doses: 97 mm (R33) and 194 mm (R66). For the three other rainfall conditions, we artificially increased rainfall in unsheltered plots using a watering pot: 295 mm (CK), 392 mm (R133), and 490 mm (R166). The temperature consisted of two levels: the actual temperature (CK) and the interaction between rainfall and the temperature, which was increased by about 2 °C (T) with the OTC (Open-Top Chamber) in each plot. TR33 was the first site of interaction between 33% precipitation (R33) and the temperature increase of about 2 °C (T), and the marks of TR66, TCK, TR133, TR166 were the same. R33 was the first site of 33% precipitation, and the marks of R66, CK, R133, R166 were the same.

In both the fungi and bacteria communities, *α*-diversity increased with increased temperature (Figure 7).

### 2.7. Soil Bacteria and Fungi β-Diversity

**Figure 8:**
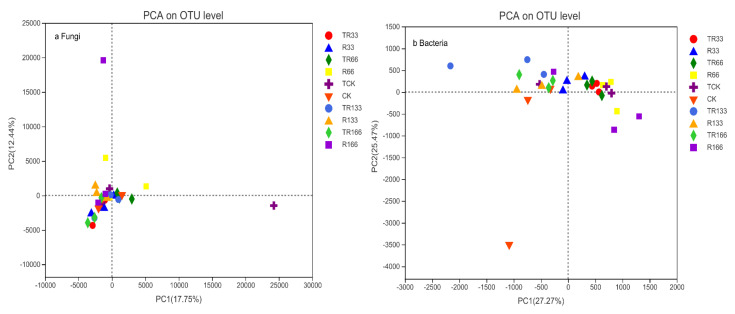
Soil microorganism *β*-diversity of (**a**) fungi and (**b**) bacteria in the study sites by principal component analysis (PCA). Five levels of rainfall (R) were used: 33% (R33), 66% (R66), 100% (CK), 133% (R133), and 166% (R166) of the annual average. The first two rainfall conditions were obtained by using two rainout shelters with two manipulated rainfall doses: 97 mm (R33) and 194 mm (R66). For the three other rainfall conditions, we artificially increased rainfall in unsheltered plots using a watering pot: 295 mm (CK), 392 mm (R133), and 490 mm (R166). The temperature consisted of two levels: the actual temperature (CK) and the interaction between rainfall and the temperature, which was increased by about 2 °C (T) with the OTC (Open-Top Chamber) in each plot. TR33 was the first site of interaction between 33% precipitation (R33) and the temperature increase of about 2 °C (T), and the marks of TR66, TCK, TR133, TR166 were the same. R33 was the first site of 33% precipitation, and the marks of R66, CK, R133, R166 were the same.

In the fungi communities, the distance between each sample point was the farthest under TCK, and the distance was 25124 according to PCA; therefore, the corresponding *β*-diversity was the highest under TCK. In the bacteria communities, the distance between each sample point was the farthest under CK; therefore, the corresponding *β*-diversity was the highest under CK, and the distance was 3010 according to PCA (Figure 8).

### 2.8. The Relationship between Grassland Plant Diversity, Biomass, Soil Bacteria, and Fungi α- and β-Diversity

**Figure 9:**
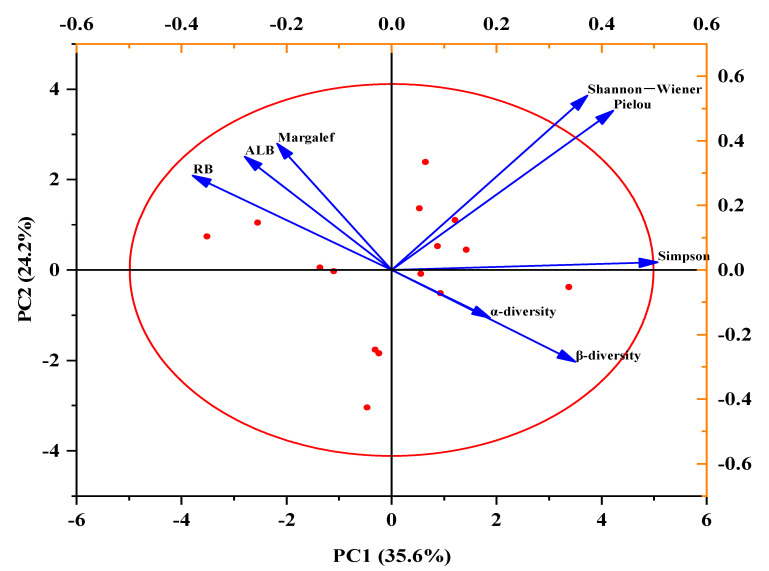
Principal components analysis (PCA) plots showing the influence of the Shannon–Wiener, Pielou, Margalef, and Simpson indexes, and the above-living biomass (ALB), root biomass (RB), *α*-diversity, and *β*-diversity, which represented effects of different temperature (recorded as CK and T) and variation in precipitation (recorded as R33, R66, CK, R133, R166).

The Shannon–Wiener diversity index was positively correlated with the ALB, *α*-diversity, and *β*-diversity but negatively correlated with the RB. The Pielou evenness index was positively correlated with *α*-diversity and *β*-diversity but negatively correlated with the ALB and RB. The Margalef species richness index was positively correlated with the RB and ALB. The Simpson dominance index was positively correlated with *α*-diversity and *β*-diversity but negatively correlated with the RB and ALB. The RB was greatly negatively correlated with *β*-diversity (Figure 9).

## 3. Discussion

### 3.1. Effects of Precipitation Changes and Temperature on Plant Main Value

Under the changing precipitation condition and the interaction of the changing precipitation and the increasing temperature conditions, the main values of *Agropyron mongolicum*, *Lespedeza bicolor*, and *Polygala tenuifolia* were all higher than the other plants, so we made sure that the three plants were dominant plants; the reason might have been because the root system of *Lespedeza bicolor* forms vertical and horizontal networks in the soil layer, helping it to make full use of the water and nutrients therein. Furthermore, the proportion of woody and sclerenchyma cell tissue in Mongolia wheatgrass is large, and the roots of *Polygala tenuifolia* are strong and can absorb water better. Thus, these characteristics of the three plants give them better drought resistance than other species.

### 3.2. Effects of Precipitation Changes and Temperature on Plant α-Diversity

Species diversity directly affects ecosystem function and stability, which is the foundation of human survival and development [19]. The distribution pattern of species diversity and its influencing factors have become the core problem of ecology and biogeography research [20].

The Shannon–Wiener and Simpson in CK and TCK are significantly higher than other treatments. This might have been because the sparse surface vegetation, loose soil structure, and low water-holding capacity of desert steppe. Precipitation directly affects the soil moisture content. The decrease of precipitation leads to the drought of topsoil and thus directly reduces the effective moisture content in soil. The change of soil moisture content indirectly affects soil nutrients and indirectly affects the absorption, transportation, and utilization of nutrients by plants by limiting the normal activities of rhizosphere microorganisms, resulting in the reduction of plant species. When precipitation increases, soil erosion occurs, and the reduction of carbon, nitrogen, and other nutrients in soil limits the growth of plants. The distribution pattern of species diversity and its influencing factors have become the core problem of ecology and biogeography research.

### 3.3. Effects of Changing Precipitation and Increasing Temperature on the Number of Species

With increased precipitation, the number of species was highest under R166, possibly because plant roots need to absorb more water to grow in desert grasslands. This finding agrees with a previous study that found fine roots may have complex responses to the higher amount of precipitation predicted for the future [21].

### 3.4. Effects of Changing Precipitation and Increasing Temperature on Plants and Dominant Species Biomass

This study found that increased precipitation promoted the growth of RB more than ALB, but the effect of rising temperature on RB was not clear. When a plant is subjected to drought stress, it will reduce ALB and increase underground RB. Some studies have found that reducing drought stress caused by a lack of precipitation will prompt plant to allocate more biomass to the underground RB, so that the root system can better absorb water and nutrients in deep soil [22]. Plant biomass increases with rising precipitation, probably because increased precipitation can effectively supplement soil moisture and promote plant growth and development.

With increasing precipitation, R166 was found to promote the ALB and total biomass of *Agropyron mongolicum* the most, and R33 promoted the ALB, RB, and total biomass of *Lespedeza bicolor* the most. The ALB, RB, and total biomass of *Polygala tenuifolia* was highest under natural precipitation. Under rising temperatures, with increased precipitation, R166 was found to promote the total biomass of *Agropyron mongolicum* the most, and R33 promoted the total biomass of *Lespedeza bicolor* the most. *Polygala tenuifolia* had the highest biomass under natural precipitation, possibly because *Agropyron mongolicum* is a graminoid plant and needs to absorb more water than *Polygala tenuifolia* (*Leguminosae* plant) and *Lespedeza bicolor* (*Lespedeza bicolor* plant).

### 3.5. Effects of Changing Precipitation and Increasing Temperature on Plant and Dominant Species Organic Carbon, Total Nitrogen, and Total Phosphorus

Under rising temperature with increasing precipitation, the differences in plant nutrients were not obvious. TR promoted plant organic carbon, nitrogen, and phosphorus content more than R, possibly because higher temperatures promote root respiration, and the root absorbs more elements from soil through active transpiration. This accords with a previous study that indicated that roots were the main source of SR, which contributed >70% of CO_2_ emissions [23].

### 3.6. Effects of Changing Precipitation and Increasing Temperature on Soil Bacteria and Fungi Diversity

According to a recent study, compared with soil bacteria and fungi biomass and activity, community structure is more sensitive to warming, with seasonal changes in temperature found to have a significant impact on soil bacteria and fungi communities [24]. Our research found that changes in precipitation and increased temperature promoted fungi communities but did not have a significant effect on the bacteria communities. In fungi communities, TCK promoted the most *β*-diversity, but in the bacteria communities, CK promoted the most *β*-diversity. Increasing temperatures may provide a more suitable growth environment for fungi by affecting the availability of plant litter components and nutrients. Therefore, increasing temperature may be beneficial to the growth of fungi and may inhibit bacteria growth, thereby changing bacteria and fungi community structure. For desert grassland, an increase in temperature will also change soil temperature and moisture levels, which may further change the diversity of soil bacteria and fungi communities.

### 3.7. Effects of Precipitation Changes and Temperature on the Relationship between Grassland Vegetation Diversity, Biomass, and Soil Bacteria and Fungi Diversity

Soil bacteria and fungi *α*- and *β*-diversity and ALB all promoted plant diversity, but RB had a limited function. The soil bacteria and fungi communities were able to promote the decomposition of soil nutrients, offering more nutrients for plant to absorb and allowing more different kinds of plants to grow. This is consistent with previous research [25].

## 4. Materials and Methods

### 4.1. Study Site

**Figure 10:**
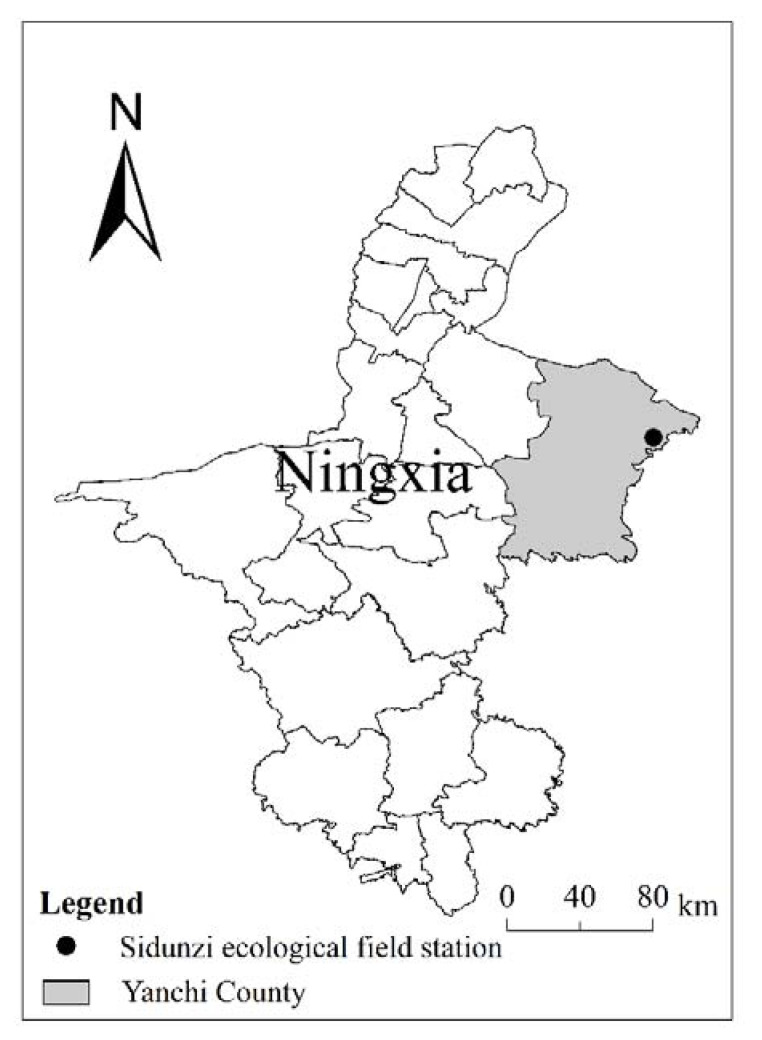
Location of the Sidunzi Village of Ningxia Observatory on the Loess Plateau.

The study area was located in the desert steppe of the Sidunzi ecological field station of Ningxia in China (37°47′ N 107°25′ E), which is on the southern edge of the Mu Us Sandy land and the yellow transition zone from the soil plateau to the Ordos platform (Figure 10). The natural conditions are relatively poor, characterized by drought, low rainfall, and strong winds and storms, and the region has typical temperate continental monsoon climate. Its annual average temperature is 8.1 °C, its monthly average temperature is −13.0–22.7 °C, its extreme maximum temperature was 34.9 °C, its extreme minimum temperature was −24.2 °C, its annual average frost-free period is 162 days, and its annual average precipitation is less than 300 mm. Its zonal soil structure is loose, and its soil fertility is low. Its zonal vegetation is typical of a desert steppe, and its dominant plants are Agropyron mogolicum Keng, Lespedeza potaninii Vass, and Polygala tenuifolia Willd. Due to the influence of climatic conditions and human activities, the grassland in the region has been degraded in large areas for a long time.

### 4.2. Experimental Design

We completed all control experiment devices from June 2018 to March 2019. We started our experiment in May 2019 and collected soil and plant samples from July 2019.

**Figure 11:**
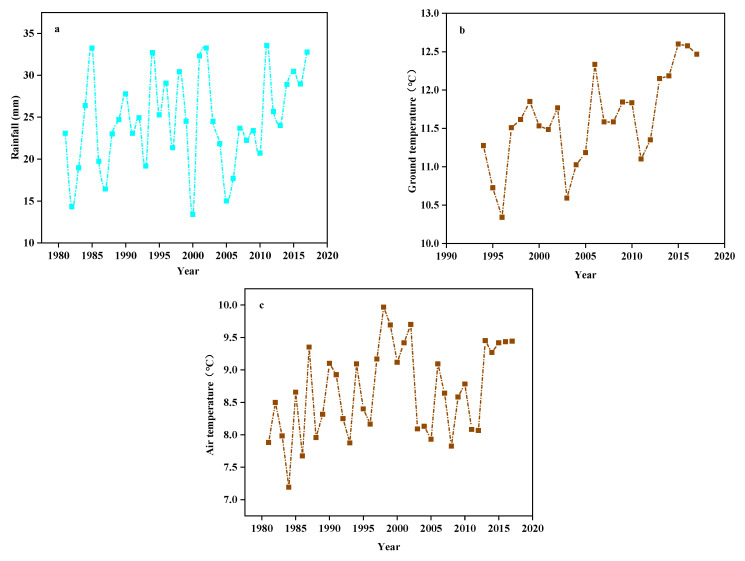
The (**a**) Rainfall, (**b**) Ground temperature, and (**c**) Air temperature from 1981 to 2017.

According to meteorological monitoring of the study site from 1981 to 2017, its annual average precipitation, ground temperature, and air temperature all showed rising trends (Figure 11). Artificial rain-collecting greenhouses and sprinkler irrigation techniques were used to achieve 66% and 133% precipitation gradients and to ensure the precipitation treatment was kept within the 37-year average precipitation and fluctuation extremes. We established two temperature increase gradients to reflect the steady increases in ground temperature and air temperature recorded by meteorological monitoring.

**Figure 12:**
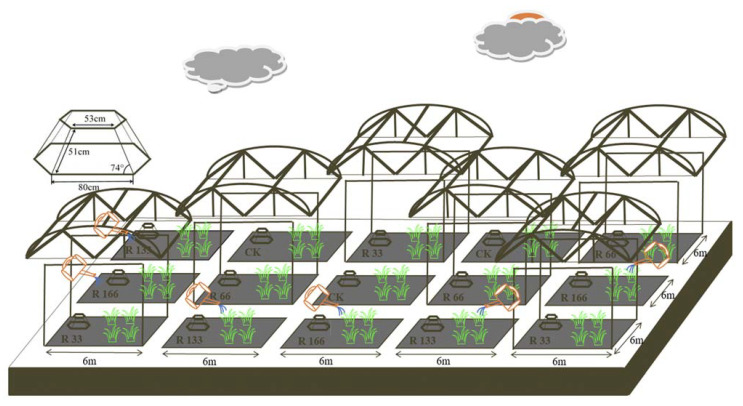
Rain shelter construction and Open-Top Chamber (OTC) arrangements of the subplots at the study sites. Five levels of rainfall (R) were used: 33% (R33), 66% (R66), 100% (CK), 133% (R133), and 166% (R166) of the annual average. The first two rainfall conditions were obtained by using two rainout shelters with two manipulated rainfall doses: 97 mm (R33) and 194 mm (R66). For the three other rainfall conditions, we artificially increased rainfall in unsheltered plots using a watering pot: 295 mm (CK), 392 mm (R133), and 490 mm (R166). The temperature consisted of two levels: the actual temperature (CK) and the interaction between rainfall and the temperature, which was increased by about 2 °C (T) with the OTC (Open-Top Chamber) in each plot.

The designed rainout shelter was completed in November 2018, and these shelters were randomly built (Figure 12). The rainfall gradient was constructed with artificial shelters and sprinklers, and the rainout shelters were made of polycarbonate material, which can allow 90% of photosynthetic effective radiation to pass through it. A two-factor completely randomized experimental design was used based on rainfall and temperature factors. Five levels of rainfall were used: 33% (R33), 66% (R66), 100% (CK), 133% (R133), and 166% (R166) of the annual average. The first two rainfall conditions were obtained by using two rainout shelters with two manipulated rainfall doses: 97 mm (R33) and 194 mm (R66). For the three other rainfall conditions, we artificially increased rainfall in unsheltered plots using a watering pot: 295 mm (CK), 392 mm (R133), and 490 mm (R166). The temperature consisted of two levels: the actual temperature and the interaction between the rainfall and the temperature, which was increased by about 1.5 °C with the OTC (Open-Top Chamber) in each plot [26]. The OTC was made of acrylic transparent board material, which can allow 90% of photosynthetic effective radiation to pass through it. The area of each plot was 6 × 6 m, and each treatment (n = 5) was repeated three times, for a total of 15 plots (temperature treatments are included in the precipitation treatments) (Figure 3). On the 15th and 30th of each month, R33 and R66 of the natural rainfall during the 1st–15th and the 16th–30th of the month, respectively, were collected from the actual rainfall and then evenly replenished to the plots containing R133 and R166 by a watering pot.

### 4.3. Collection of Soil Microorganism Samples

In each plot, including the inner OTC, we collected 0–10 cm of soil from each sample plot. We removed the impurities in the soil, including plants, moss, visible roots, litter, and visible soil animals, and then wiped the sampler with alcohol-soaked cotton. After the alcohol had completely evaporated, we used the soil in the sample to soak the sampler. This step needed to be repeated each time the sample changed. Three points sampled from the same quadrant were mixed as one soil sample. We placed mixed soil into a 10 mL centrifuge tube and then transferred it to a −80 °C refrigerator for determination of soil microbes. We used the Operational Taxonomic Units (OTU) lever to determine the soil microorganism taxonomic group. The Operational Taxonomic Unit (OTU) is an operational definition used to classify groups of closely related individuals. In the context of numerical taxonomy, the most abundant sequence type was selected to represent each OUT [27]. In our research, OTUs are in the absence of traditional systems of biological classification (which are available for macroscopic organisms), pragmatic proxies for “species” (microbial). For several years, OTUs have been the most commonly used units of diversity, especially when analyzing small subunit 16S for prokaryotes (as is the case of this work bacteria) or 18S (fungi) marker gene sequence datasets [28].

### 4.4. Collection of Plant Samples

The measurement of community *α*-diversity can be divided into the Shannon–Wiener diversity index, Margalef species richness index, Pielou evenness index, and Simpson dominance index. These four commonly used *α*-diversity indexes incorporate two measurements, the number (richness) of species, and the uniformity of species.

The richness index mainly measures the number of species within a certain spatial range to express the richness of organisms; the evenness index is a single statistic that combines the richness index and the evenness index; and the diversity index is based on the number of species to reflect a community’s diversity, which can describe the disorder and uncertainty of an individual species. An increase in the number of species in the community represents an increase in the community’s complexity. The greater the index value, the greater the amount of information contained in the community [9]. The importance value (IV) is calculated using the relative density, relative frequency, relative coverage, relative height, and relative biomass, according to the following formula:

Importance value (IV) = (Relative density + relative coverage + relative frequency + relative height + relative biomass)/5

We calculated plant diversity in terms of the number of species in each plot(s), the relative importance of the species in the plot (Pi), and the number of individuals in all species (N), according to the following formula:

Shannon–Wiener diversity index: $H=-\sum_{i=1}^{s}\mathrm{PilnPi}$
Margalef species richness index: R = (S − 1)/$\log_{10}N$
Pielou evenness index: E = H/lnS
Simpson dominance index: C = 1 − ∑(Pi)^2^

Dominant plant species: We measured the plant relative biomass, relative height, relative cover, relative frequency, and relative density to determine the importance values, and then used importance values to determine the dominant species.

Plant carbon, nitrogen, and phosphorus: Plant organic carbon and total carbon were measured with a total organic carbon (TOC) analyzer (CS Analysis Instrument, Naples, FL, USA), and total nitrogen and total phosphorus were measured with a HCLO_4_-H_2_SO_4_ digestion-flow injection instrument (model Skalar-SAN++, Delft, The Netherlands).

Litter: We picked up the litter on the ground by hand in the sample squares that cut off the ground plants and carefully removed the fine soil particles attached to the litter and put them in the envelopes according to the sample squares. We dried samples at 65 °C to constant weight, weighed them, and recorded the dry weight data.

Plant height: The natural height of different plants in each sample box was measured 5 times, respectively. If there were not 5 plants, the plants outside the sample box were selected for measurement.

Plant coverage: The acupuncture method was used. A 1 m^2^ square sample rope was placed on the ground and divided into an average of 100 grids. Plants were acupunctured in order every 10 cm from top to bottom and from left to right with a 2 mm needle. If the needle contacted with the plant, it was counted as 1, and it was not counted if there was no contact. The coverage of each plant in the sample was not more than 100. If two plants occurred simultaneously during acupuncture, the total coverage was reduced by 1, and if three plants occurred simultaneously, the total coverage was reduced by 2. (Note: Total coverage is the sum of coverage of each plant.)

Plant frequency: Rounds were thrown 10–15 times in the plot, and the total number of times that each plant appeared was the frequency of the plant. (Note: In each round thrown, as long as the plant appeared, regardless of the number of plants, the frequency was 1). The total frequency was the sum of the frequency of each plant.

Plant density: The kinds of plants and the number of times each plant appeared in the sample box were recorded.

Plant biomass: We measured plant biomass in a 1 m^2^ quadrat that was randomly selected in each plot at the end of July 2019. We dug all plants in each plot out from the soil. We then cut the aboveground living plant and plant roots separately while sorting them according to species and placed them into respective envelopes. These species were then taken into the laboratory and dried at 65 °C in the oven for 48 h; the aboveground living plant biomass (ALB) and plant root biomass (RB) were then calculated.

### 4.5. Statistical Analysis

We used repeated-measures ANOVA to examine the differences in the plant *α*-diversity index, the number of species and plants and the dominant species of the biomass, the organic carbon, total nitrogen, and total phosphorus of the plants and the dominant plant species under different precipitation levels and the interaction between precipitation and temperature by SPSS 21.0. *t*-tests were used to examine the differences in the plant *α*-diversity index, the number of species and plants and the dominant species of biomass, and the organic carbon, total nitrogen, and total phosphorus of plants and dominant plant species under different temperatures by SPSS 21.0. The soil microorganism *β*-diversity was analyzed by principal component analysis (PCA) using R. We used principal components analysis (PCA) to examine the relationships between the grassland plant diversity, biomass, and soil bacteria and fungi *α-* and *β*-diversity by Origin 2021.

## 5. Conclusions

In this study, plant *α*-diversity in CK and TCK under the altered precipitation were significantly higher than other treatments. Under the interaction of the increasing precipitation and the rising temperature conditions, R166 promoted the number of species the most. Increasing precipitation was found to promote the growth of RB more than ALB, but the effect of rising temperatures on RB was not clear. The changing precipitation and increasing temperature factors, and the interaction of the two factors, all had no significant impact on the biomass, organic carbon, total nitrogen, and total phosphorus of plants. R166 promoted the ALB, RB, and total biomass of Agropyron mongolicum the most. The TR treatment promoted plant organic carbon, nitrogen, and phosphorus content more than R. In the fungi communities, under rising temperature, increasing precipitation promoted *α*-diversity, but *α*-diversity did not obviously vary in the bacteria communities. In the fungi communities, TCK promoted the most *β*-diversity, but in the bacteria communities, CK promoted the most *β*-diversity. Soil bacteria and fungi *α*- and *β*-diversity, and ALB promoted plant diversity the most.

## Figures and Tables

**Table 1:** Grassland plant *α*-diversity index under different precipitation and temperature treatments.

	Shannon–Wiener	Pielou	Margalef	Simpson
	F = 6.12 *p* > 0.05	F = 4.84 *p* > 0.05	F = 6.34 *p* > 0.05	F = 3.71 *p* > 0.05
R33	1.64 ± 0.15 a	0.80 ± 0.03 a	3.17 ± 0.76 a	0.76 ± 0.05 a
R66	1.65 ± 0.07 a	0.75 ± 0.04 a	3.75 ± 0.39 a	0.75 ± 0.03 a
CK	1.80 ± 0.11 a	0.83 ± 0.04 a	3.30 ± 0.35 a	0.79 ± 0.03 a
R133	1.70 ± 0.07 a	0.85 ± 0.05 a	2.84 ± 0.21 a	0.77 ± 0.04 a
R166	1.68 ± 0.11 a	0.73 ± 0.07 a	3.70 ± 0.39 a	0.76 ± 0.03 a
	F = 6.09 *p* > 0.05	F = 4.79 *p* > 0.05	F = 5.98 *p* > 0.05	F = 3.69 *p* > 0.05
TR33	1.65 ± 0.14 a	0.82 ± 0.04 a	3.18 ± 0.74 a	0.77 ± 0.03 a
TR66	1.67 ± 0.06 a	0.76 ± 0.03 a	3.76 ± 0.38 a	0.76 ± 0.02 a
TCK	1.81 ± 0.10 a	0.84 ± 0.03 a	3.31 ± 0.37 a	0.80 ± 0.02 a
TR133	1.71 ± 0.08 a	0.86 ± 0.06 a	2.85 ± 0.23 a	0.78 ± 0.03 a
TR166	1.69 ± 0.10 a	0.75 ± 0.06 a	3.71 ± 0.40 a	0.77 ± 0.02 a

Mean ± SE followed by lowercase letters in each column indicates significant differences between the variance percentage of precipitation, according to LSD test (*p* < 0.05). Five levels of rainfall (R) were used: 33% (R33), 66% (R66), 100% (CK), 133% (R133), and 166% (R166) of the annual average. The first two rainfall conditions were obtained by using two rainout shelters with two manipulated rainfall doses: 97 mm (R33) and 194 mm (R66). For the three other rainfall conditions, we artificially increased rainfall in unsheltered plots using a watering pots: 295 mm (CK), 392 mm (R133), and 490 mm (R166). The temperature consisted of two levels: the actual temperature (CK) and the interaction between rainfall and the temperature, which was increased by about 2 °C (T) with the OTC (Open-Top Chamber) in each plot. TR33 was the first site of interaction between 33% precipitation (R33) and the temperature increase of about 2 °C (T), and the marks of TR66, TCK, TR133, TR166 were the same. R33 was the first site of 33% precipitation, and the marks of R66, CK, R133, R166 were the same.

## Data Availability

Not applicable.
